# Correction: Photoactive, antimicrobial CeO_2_ decorated AlOOH/PEI hybrid nanocomposite: a multifunctional catalytic-sorbent for lignin and organic dye

**DOI:** 10.1039/d0ra90136a

**Published:** 2020-12-17

**Authors:** K. Aboo Shuhailath, Vazhayal Linsha, Sasidharan Nishanth Kumar, K. Babu Babitha, Abdul Azeez Peer Mohamed, Solaiappan Ananthakumar

**Affiliations:** Academy of Scientific and Innovative Research [AcSIR] Ghaziabad-201002 India; Functional Materials Section, Materials Science and Technology Division (MSTD), National Institute for Interdisciplinary Science and Technology (CSIR-NIIST) Trivandrum-695 019 Kerala India ananthakumar70@gmail.com +91-471-2515289; Agroprocessing and Natural Products Division, National Institute for Interdisciplinary Science and Technology (CSIR-NIIST) Trivandrum-695 019 Kerala India

## Abstract

Correction for ‘Photoactive, antimicrobial CeO_2_ decorated AlOOH/PEI hybrid nanocomposite: a multifunctional catalytic-sorbent for lignin and organic dye’ by K. Aboo Shuhailath *et al.*, *RSC Adv.*, 2016, **6**, 54357–54370, DOI: 10.1039/C6RA07836B.

The authors regret that the one of the affiliations (affiliation *a*) was incorrectly shown in the original manuscript. The corrected list of affiliations is as shown above.

The Royal Society of Chemistry apologises for these errors and any consequent inconvenience to authors and readers.

## Supplementary Material

